# Immunology repertoire study of pulmonary sarcoidosis T cells in CD4+, CD8+ PBMC and tissue

**DOI:** 10.18632/oncotarget.20085

**Published:** 2017-08-09

**Authors:** Yingyun Fu, Yazhen Li, Lan Xu, Shengguo Liu, Minlian Wang, Lu Xiao, Song Liu, Yong Dai

**Affiliations:** ^1^ Department of Respiratory and Critical Care Medicine, The Second Medical College of Jinan University, Shenzhen People's Hospital, Luohu District, Shenzhen, China; ^2^ Clinical Medical Research Center, The Second Clinical Medical College of Jinan University, Shenzhen People's Hospital, Luohu District, Shenzhen, China; ^3^ Shenzhen Key Laboratory of Respiratoty Disease, Shenzhen, China

**Keywords:** sarcoidosis, immune system, CDR3, TCR BV CDR3, PMBCs, Immunology and Microbiology Section, Immune response, Immunity

## Abstract

Sarcoidosis is a systemic granulomatous disorder highly related with immune response. The diversity and stability of the immune system could be measured by hypervariable complementarity-determining region 3 (CDR3) segments of the T cell receptor (TCR). Here we used a combination of multiplex PCR and next-generation sequencing to conduct a good quality analysis of the T-cell receptor BV complementarity-determining region 3 (TCR BV CDR3) gene in peripheral blood mononuclear cells (PBMCs) from 7 sarcoidosis patients and lung sarcoidosis tissue from 6 patients. The length distribution of CDR3 sequences identified a significant difference among CD4+, CD8+ and tissue samples. The analysis of Gini coefficient, Shannon entropy and HEC number showed that they all presents in sarcoidosis tissue group clones in a more skewed manner than that of in PMBCs groups. 2 nucleotide sequences and 2 amino acid sequences were shared by all samples. The comparison of TRBV, TRBJ usage and VJ combination frequency identified 2 TRBV genes, 2 TRBJ genes differentially expressed among different groups and different higher usage and lower usage of V-J combinations between each group.

## INTRODUCTION

Sarcoidosis is a systemic granulomatous disorder of unknown etiology that affects patients all around the world [1]. It is characterized by the accumulation of activated and proliferating T-Iymphocytes and macrophages in the involved organs, most commonly in the lung [2]. A history of present illness should elicit pulmonary symptoms of sarcoidosis, including cough, dyspnea or chest pain. Involvement of lung is accounting for over 90% of cases [3]. The diagnosis of sarcoidosis, is based on a compatible clinical-radiological picture and the histological evidence of non-caseating granulomas. Lung and thoracic lymph nodes are the key organs for the clinical diagnosis [4]. The presentations of pulmonary sarcoidosis may vary from a radiographic abnormality detected in an asymptomatic individual to a progressive pulmonary disorder causing lung fibrosis and respiratory failure. It is problematic to determine the frequencies of the various presentations and natural courses of sarcoidosis because they vary according to the population that is examined. Radiographic screening studies tend to yield higher incidence rates of sarcoidosis by detecting more asymptomatic cases [5]. Because Africans tend to have more severe manifestations of sarcoidosis than Caucasus or Asians [6], populations with different percentage of various races have different frequencies and pattern of disease presentations.

The immune pathogenesis of sarcoidosis is not completely understood, but there has been tremendous progress in the past decade. The pathogenesis of sarcoidosis seems to involve the interplay of antigen, HLA class II molecules, and T-cell receptors [7]. Investigation of the genes associated with sarcoidosis has focused on the HLA genes [5]. Genome-wide approaches have identified non-HLA candidate susceptibility genes. For example, a family-based study among sarcoidosis-affected German families led to the discovery of a mutation in a putative immune regulatory gene, butyrophilin-like 2 (BTNL2), that may explain 23% of the attributable risk in that population [8]. The granuloma in sarcoidosis is characterized by a core of monocyte-derived epithelioid histocytes and multinucleate giant cells with interspersed CD4+ T lymphocytes. However, CD8+T lymphocytes, fibroblasts, regulatory T cells, and B lymphocytes are present within the granuloma. The T-cell response is biased toward a Th1 phenotype, with important roles for IFN-g and interleukin-12 [7]. A variety of chemokines and cytokines have been associated with the granulomatous response in sarcoidosis, including tumor necrosis factor a (TNF-a) [9, 10]. The importance of TNF in sarcoidosis has been validated by studies documenting effectiveness of biologic TNF antagonists in sarcoidosis in treating some patients with sarcoidosis. In the present study, we conducted immunology repertoire study of pulmonary sarcoidosis T cells in CD4+, CD8+ PBMC and sarcoidosis tissue to reveal the immunological affect in sarcoidosis.

## RESULTS

### Sequence data

To study the characteristics of immune repertoire for different tissue types of sarcoidosis disease, 7 patients were recruited. We sequenced CD4+ cell group (*n* = 7), CD8+ cell group (*n* = 7) and lung sarcoidosis tissue group (*n* = 6, one patient failed to provide tissue), all sequences were aligned to TCR references from IMGT database and deeply analyzed for further study. Within the 3 groups studied, we filtered the adapter sequences, contamination and low quality reads, the Q20 of the data is more than 80%. All together there were 12,635,182 sequencing reads and 631759.1 sequencing reads on average. The mean unknown sequences number (reads = 7,259.3), productive sequences number (reads = 432,617.55), Non_productive sequences number (reads = 191,882.25), In_frame sequences number (458,987.35) and Out-of_frame sequences number (reads = 162,668.7) per sample were listed in Table 1. There were 416,282.55 total CDR3 sequences number, 19,892.9 unique CDR3 nucleotide sequences number and 16,525.95 unique CDR3 amino acid sequences number per sample as listed in Table 1.

**Table 1:** Sequencing quality data of all samples

Sample ID	Total reads	immune sequences number	Unknown sequences numebr	productive sequences number	Non_productive sequences number	In-frame sequences number	Out-of_frame sequences number	Total CDR3 sequences number	Unique cdr3 nt sequences number	Unique cdr3 aa sequences number
1-CD4	982482	977713	4769	780923	196790	812791	162802	768159	44863	36275
1-CD8	1019665	1010032	9633	651284	358748	681950	323796	635130	30211	24347
1-Tissue	826801	814689	12112	564771	249918	579149	232756	549839	11864	8861
2-CD4	875416	871213	4203	720687	150526	744888	124171	709860	25395	19812
2-CD8	804580	799020	5560	478189	320831	513158	283181	454400	21587	18177
2-Tissue	264682	257618	7064	131241	126377	136759	117761	126659	5849	4668
3-CD4	629771	626450	3321	493196	133254	514152	110960	477407	34832	28588
3-CD8	673637	667934	5703	507676	160258	534196	131185	490002	25857	20706
3-Tissue	173346	166956	6390	85734	81222	88235	77378	36817	1138	890
4-CD4	636166	613326	22840	334755	278571	351373	254623	294852	10897	8461
4-CD8	504161	500700	3461	377054	123646	387921	110862	371353	10538	8835
4-Tissue	875950	870778	5172	612724	258054	636158	231659	603077	8563	6150
5-CD4	529970	525726	4244	377036	148690	389611	133652	370826	35632	33259
5-CD8	684006	680876	3130	530370	150506	544488	134645	525921	34633	31673
5-Tissue	276040	268304	7736	170723	97581	175980	89388	160569	8113	6464
6-CD4	457009	454886	2123	359367	95519	375971	77831	353271	44057	38882
6-CD8	411422	407853	3569	325237	82616	336866	68811	316420	9609	7364
6-Tissue	423421	409533	13888	247874	161659	254589	150408	227670	3990	3273
7-CD4	805096	801055	4041	527367	273688	548346	250976	510035	22370	17668
7-CD8	781561	765334	16227	376143	389191	573166	186529	343384	7860	6166
Average	631,759.10	624,499.80	7,259.30	432,617.55	191,882.25	458,987.35	162,668.70	416,282.55	19,892.90	16525.95

### CDR3 length distribution mode analysis

**Figure 1:**
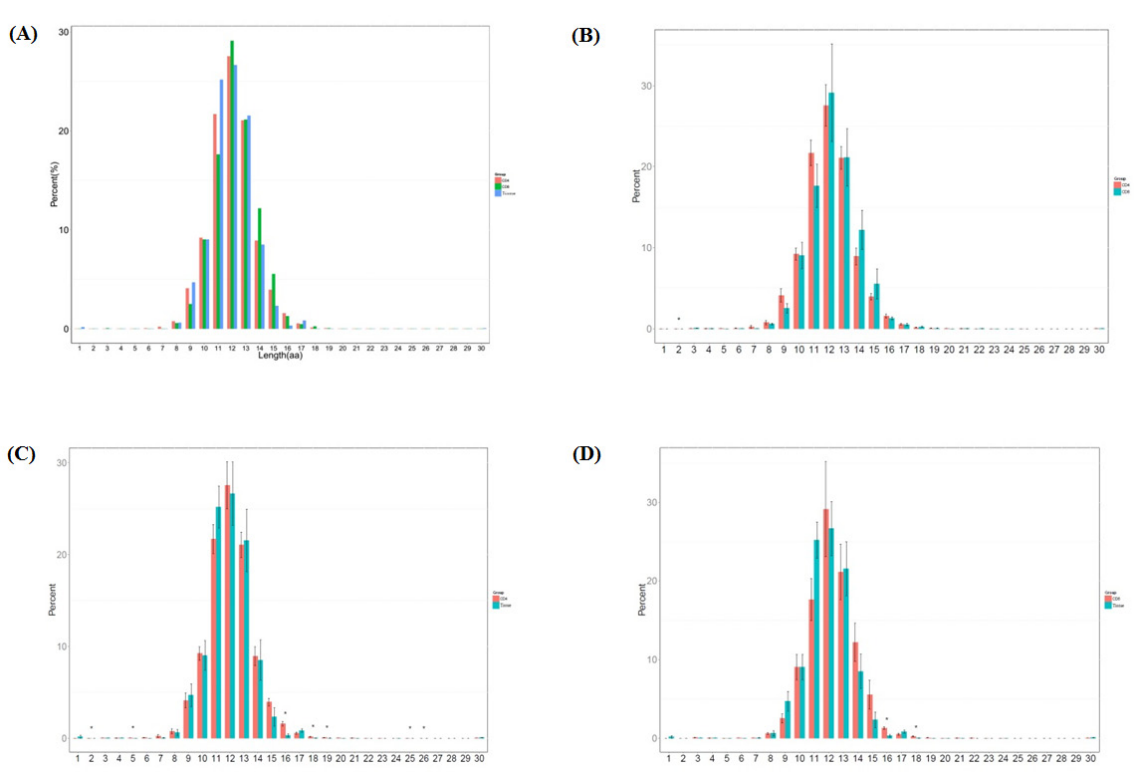
Length distribution of CDR3 sequences (aa) in the CD4+ group, CD8+ group and tissue group **A.** The different percentage (%) of each amino acid length in 3 groups. **B.** The different percentage (%) of each amino acid length compared between CD4+ and CD8+ groups (*p* < 0.0061). **C.** The different percentage (%) of each amino acid length compared between CD4+ and tissue groups (*p* < 0.003, *p* < 0.047, *p* < 0.0011, *p* < 0.0012, *p* < 0.013, *p* < 0.034, *p* < 0.033). **D.** The different percentage (%) of each amino acid length compared between CD8+ and tissue groups (*p* < 0.0022, *p* < 0.018).

CDR3 length is an important measurement for TCR repertoire diversity. In this study, the length distribution of CDR3 in three groups was first analyzed. The percentage of amino acid length 2 was significantly higher in CD4+ group than in CD8+ group (*p* < 0.0061). The percentage of amino acid length 2 (*p* < 0.003), 5(*p* < 0.047), 16 (*p* < 0.0011), 18 (*p* < 0.0012), 19 (*p* < 0.013), 25 (*p* < 0.034), 26 (*p* < 0.033) were significantly higher in CD4+ group compared to sarcoidosis tissue group. And the percentage of amino acid length 16 (*p* < 0.0022), 18 (*p* < 0.018) were significantly higher in CD8+ group compared to tissue group, shown in Figure 1.

In normal mode, the CDR3 amino acid length distribution should display Gaussian distribution. As mentioned above, we compared the distribution of CDR3 amino acid length. We then fit the Gaussian distribution of each sample and compared the R^2 value between each sample and each group. The R^2 value ranged from 0 to 1, suggests the worst fitted Gaussian distribution to best distribution. According to R^2 value the length distribution of CD4+ group is better fitted to Gaussian distribution and the tissue group is worse fitted (no Sig.), show in Supplementary Figure 1.

### Gini coefficient, Shannon entropy and HEC analysis

**Figure 2:**
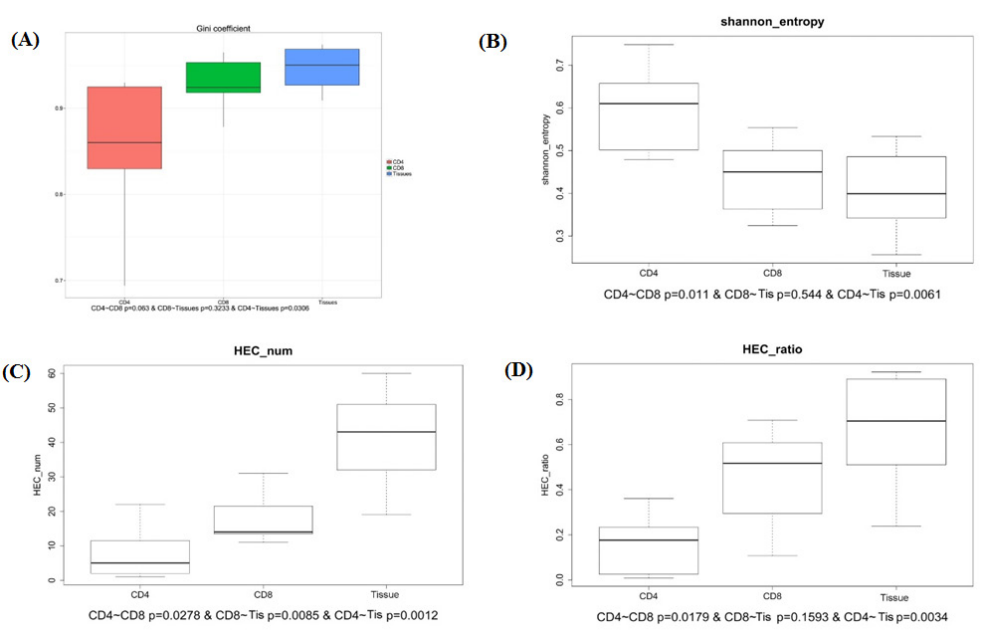
Comparison of Gini coefficient, Shannon entropy, HEC number, HEC ratio in CD4+, CD8+ and tissue groups **A.** The comparison of Gini coefficient in 3 groups shows significant different. **B.** The comparison of Shannon entropy in 3 groups. **C.**, **D.** The comparison of HEC number and HEC ration in 3 groups.

To study the diversity and stability of patients’ immune system, further study of sequence distribution and clone expansion were analyzed. We calculated the Gini coefficient of CD4+ group, CD8+ group and sarcoidosis tissue group and found the tissue group with the highest Gini coefficient, CD4+ group the lowest and medium CD8+ group (Figure 2). The difference between CD4+ group and tissue group is significant (*p* < 0.03). This result indicated CD4+ group has the most evenly distributed sequences which are related to a highest TCR diversity. On the contrary the tissue group sequences distributed most unevenly and related to the lowest TCR diversity.

Another statistic metric was also introduced to observe the TCR diversity in 3 groups. The Simpson index measures the sample richness and the degree of uniformity in clone frequencies. The entropy value reflects the diversity distribution of CDR3 clones. It ranges from 0 to 1, the most diversity of immune system situation is measured 1, which means every sequence is represented once, the least diversity is measured by a score of 0. We calculated Shannon entropy of 3 groups, the CD4+ group has the highest entropy which is significantly different compared to both CD8+ group (*p* < 0.011) and tissue group (*p* < 0.0061), represent the highest TCR diversity. The sarcoidosis tissue group has the lowest entropy represents the lowest TCR diversity (Figure 2). The Shannon entropy analysis result is consistent with Gini coefficient, both indicate the high TCR diversity of CD4+ group and low TCR diversity of tissue group.

The expansion level of each unique clone is another major measurement for immune diversity. After aligning to the human genome reference, the expression level of each clone is calculated. In this study, the TCR clones with frequency above 0.5% of total reads in a sample were defined as high expansion clones (HECs). In CD4+ group we observed an average HEC number of 7.85 clones (range 1-22), with average HEC ratio of 15.17% (range 0.8%-22.08%). The value in CD8+ group were 18 (range 11-31) and 44.84% (range 1.71%-70.79%). While the sarcoidosis tissue group has an average HEC number of 41.33 (rang 19-60) and average HEC ratio of 66.18% (range 23.86%-92.17%). The comparison of HEC number and HEC ratio among the three groups showed the expansion of CD4+ group is significantly less expanded than CD8+ (*p* < 0.0278 and *p* < 0.0179 respectively) and is also significantly less expanded than sarcoidosis tissue group (*p* < 0.0012 and *p* < 0.0034 respectively), while the CD4+ group is least expanded and the tissue group is the most expanded group (Figure 2). These results suggest the sarcoidosis tissue has a much more skewed clonotype spectrum than CD8+ cells or CD4+ cells.

### Pattern of CDR3 sequence sharing in different groups

The characteristics of CDR3 sequence sharing among different samples and different groups present the result of immune reaction to the same pathogen of sarcoidosis. To analyze this feature, we then analyzed different clone types. According to the sequencing statistics, the TCR repertoires of the CD4+ cell group (n = 7) obtained 2.18 × 10^5 annotated nucleotide sequence reads and 1.83 × 10^5 amino acids sequence reads. The CD8+ cell group (n = 7) obtained 1.40 × 10^5 annotated nucleotide sequence reads and 1.17× 105amino acids sequences, the data of sarcoidosis tissue group is 4.0 × 10^4 annotated nucleotide sequence reads and 3.0 × 10^4 amino acids sequences respectively. Within the analyzed sequences, 5 nucleotide sequences and 4 amino acids sequences were found in all the 7 samples in CD4+ group, 4 nucleotide sequences and 4 amino acids were found in all the 7 samples in CD8+ group, 59 nucleotide sequences and 62 amino acids sequences were found in all the 7 samples in tissue group. Two of these public nucleotides were shared by all the 20 samples from different groups, while another 2 nucleotides were shared by all samples in CD4+ group and CD8+ group. However, none of the 59 nucleotide sequences in all cases of tissue group was found sharing with 5 sequences in all cases of CD4+ group or the 4 sequences in all cases of CD8+ group except for the 2 public nucleotide sequences. And 2 amino acids sequences were shared by all the 20 samples from 3 different groups and another 2 amino acids sequences were shared by all samples in CD4+ and CD8+ groups which means all the public amino acids sequences were shared between the two lymphocytes groups. However, none of the 62 nucleotide sequences in all samples of tissue group was found sharing with the 4 amino acids sequences in all cases of CD4+ group or the 4 amino acids sequences in all cases of CD8+ group, except for the 2 public amino acids sequences. A summary of these public nucleotide sequences was listed in Table 2.

**Table 2:** Public nucleotide sequences in CD4+, CD8+, Tissue groups

Group	Public Sequences (NT)	Public Sequences (AA)
Tissue	GCCAGCAGCGAGCAGGGAAGAGATACGCAGTAT	AISSDSYNEQF
	AGCCGACGT	SVDGGRQETQY
	GCCAGTAGTAGAAGAGATGGCTACACC	ATSREGAGETQY
	GCCACCAGCAGGACAGGGTTTAATGAAAAACTGTTT	ASSGTGRWETQY
	GCCAGCATCCCCTCGCCTGTTTTCCCCGGGGAGCTGTTT	ATSGIAGETQY
	GCCAGCAGTTTTGAGACAGGGGCGGAAGCTTTC	ATSGIAGEELF
	GCCAGCAGTGACTCACTAGCGGGAGGGCGGTGG	ASSTAGSVGYGYT
	GCCACCAGCCGTCGACAGGGAGTGAATTCACCCCTCCAC	ASRGTGAGKLF
	GCCAGCAGCCAAGGCGTCCAAGAGACCCAGTAC	AKLLQTGLTGELF
	GCCACCAGCAGAGAGGGGGCGGGTGAGACCCAGTAC	AIKGTGSETQY
	GCCAGCAGTGGACAACTAGCGGGGTTTCGCACAGATACGCAGTAT	ATSDFDSGLSGTQY
	GCCACCAGCGGCATAGCGGGGGAGGAGCTGTTT	ASSPRDSHGNTIY
	GCCACCAGCAAAGACAGGGGGCAAGAGACCCAGTAC	ATSYGNVQPQH
	GCCAGCAGCCCCACCCGGACTCAGGGCTACACC	SRR
	GCCAGCAGTCCCTGGGGAGGCGGTAATGAAAAACTGTTT	AIKNTGGITEAF
	GCCACCAGCTACGGGAACGTTCAGCCCCAGCAT	ASSTWTEADVLT
	GCCATCAGTGATTCGGACAGGGCTTACTACGAGCAGTAC	ASVLQGAGSGYT
	GCCATCAAAGGGACAGGCTCCGAGACCCAGTAC	ASSQGVQETQY
	GCCAGTAGCCCAGGACGCCTTAGCTCTGGAAACACCATATAT	ASIPSPVFPGELF
	GCCAGTAGCCCAACAGGGGTGGTGGCAGAGACTCAGTAC	ATSGGSYGYT
	GCCACCAGTGAT	ASSPWGGGNEKLF
	GCCATCAGCAGTGACTCCTACAATGAGCAGTTC	ASSELRTGNTGELF
	GCCAAACTCCTTCAGACAGGCCTAACCGGGGAGCTGTTT	ASKDARNNSPLH
	GCCAGCAGTGAATTGCGGACAGGGAACACCGGGGAGCTGTTT	ATSREEKLF
	GCCAGCAGCCCTCTTCCGGGACAGGGGCGCGAGCAGTAC	ASSDVLPNTEAF
	GCCAGTAGTACATGGACGGAGGCCGACGTCCTGACT	ASFFLAGGPEETQY
	GCCACCAGTGATTTGGCCAGGCATTATGGCTACACC	ATSRRQGVNSPLH
	GCCAGCAGTCGGGGACTAGCGAGGATATACGAGCAGTAC	ASSPGRLSSGNTIY
	GCCATCAGTGAGTCAAAAGGGGACACCGGGGAGCTGTTT	ASRGSNQPQH
	GCCAGCAGTGGCAACACGGGGGGCCGCTCCTACGAGCAGTAC	ASSDSLAGGRW
	GCCAGCAGCCCGTACAGGGGGGACACCGGGGAGCTGTTT	ASSGKLTEAF
	GCCAGCGTATTACAGGGGGCTGGTTCTGGCTACACC	ASADLSIYEHY
	GCCAGTAGTACAGCAGGGTCAGTAGGCTATGGCTACACC	ASSPTGVVAETQY
	GCCAGTAGTGGGAAGTTGACTGAAGCTTTC	ATSHLGHPQETQY
	GCCACCAGTGGAGGTTCGTATGGCTACACC	AISESKGDTGELF
	GCTAGTGGTTTATCCCGACTCCAAGAGACCCAGTAC	ATSRTGFNEKLF
	GCCAGTGCCGACCTTAGCATATACGAGCATTAC	ASSGNTGGRSYEQY
	GCCACCAGCAGAGATGACAGGGGACCCGAAAAACTGTTT	ASSSRWLADNEQF
	GCCAGCAGTGGGACAGGGCGATGGGAGACCCAGTAC	ATQKGAAYGYT
	GCCTGCAAGCCGGAGTCACTCGGGACCCAGTAC	ASSGQLAGFRTDTQY
	GCCAGCTTTTTCCTAGCGGGAGGGCCTGAGGAGACCCAGTAC	ASSDDGRGRRSYEQY
	GCCAGCAGGGGGACAGGGGCTGGAAAACTGTTT	ACKPESLGTQY
	GCCACCAGCGGCATAGCGGGGGAGACCCAGTAC	ASSRDRYGTGVLYTEAF
	GCCAGGGGACTAGCGGGGACTAGCACAGATACGCAGTAT	ATAGTGNTDTQY
	GCCAGTAGTATTCAAGAGACCCAGTAC	ASSPTRTQGYT
	GCCACCAGTGATTTTGATTCGGGACTTTCGGGGACCCAGTAC	ASSPYRGDTGELF
	GCCAGCAGCCCTCGGGACAGCCATGGAAACACCATATAT	ASSIQETQY
	GCCAGCAGCCGAGGGGACAACCTAGGGAATCAGCCCCAGCAT	ASSQIGGTQETQY
	GCCAGCAGTGATGTGTTACCGAACACTGAAGCTTTC	ATSDLARHYGYT
	GCCACCGCCGGGACAGGGAACACAGATACGCAGTAT	ATSKDRGQETQY
	GCCACCAGCCACTTGGGACACCCGCAAGAGACCCAGTAC	AISDSDRAYYEQY
	GCCACCAGCAGAGAGGAAAAACTGTTT	ASSRGLARIYEQY
	GCCATCAAGAATACAGGGGGCATCACTGAAGCTTTC	ATSD
	GCCAGCAGGGGTAGCAATCAGCCCCAGCAT	ASSEQGRDTQY
	GTGCCTGAGTATGGTACAGCTAATGGCCGTCTTCATTTCCATGCGGTGCACTTTATGCGGACACTTCCTACAGGTAGCGTTGACCCTAAT	ASSPLPGQGREQY
	GCCACTCAGAAGGGGGCCGCCTATGGCTACACC	ASGLSRLQETQY
	GCCAGCAGCTCCCGGTGGCTAGCGGACAATGAGCAGTTC	ASSFETGAEAF
	GCCAGCAGTGATGATGGGCGGGGCCGCCGCTCCTACGAGCAGTAC	ASSRGDNLGNQPQH
	GCCAGCAAGGACGCCAGGAATAATTCACCCCTCCAC	VPEYGTANGRLHFHAVHFMRTLPTGSVDPN
		ATSRDDRGPEKLF
		ARGLAGTSTDTQY
		ASSRRDGYT
CD8+	AGCCGACGT	ASS
	AGCCGACGTTTTGGCGGCGCAACCTGTGACGACAAATCTGCTCAAATTTATGCGCGCTTCGATAAAAATGATTGGCGTATCCAACCTGCAGAGTTTTATCGCTTCCATGACGCAGAAGTTAACACT	SRR
	GCCAGCAGC	SRRFGGATCDDKSAQIYARFDKNDWRIQPAEFYRFHDAEVNT
	GTGCCTGAGTATGGTACAGCTAATGGCCGTCTTCATTTCCATGCGGTGCACTTTATGCGGACACTTCCTACAGGTAGCGTTGACCCTAAT	VPEYGTANGRLHFHAVHFMRTLPTGSVDPN
CD4+	AGCCGACGT	ASS
	AGCCGACGTTTTGGCGGCGCAACCTGTGACGACAAATCTGCTCAAATTTATGCGCGCTTCGATAAAAATGATTGGCGTATCCAACCTGCAGAGTTTTATCGCTTCCATGACGCAGAAGTTAACACT	SRR
	GCCAGCAGT	SRRFGGATCDDKSAQIYARFDKNDWRIQPAEFYRFHDAEVNT
	GCCAGCAGC	VPEYGTANGRLHFHAVHFMRTLPTGSVDPN
	GTGCCTGAGTATGGTACAGCTAATGGCCGTCTTCATTTCCATGCGGTGCACTTTATGCGGACACTTCCTACAGGTAGCGTTGACCCTAAT	

### Comparison of TRBV and TRBJ usage in CD4+, CD8+, tissue groups

**Figure 3:**
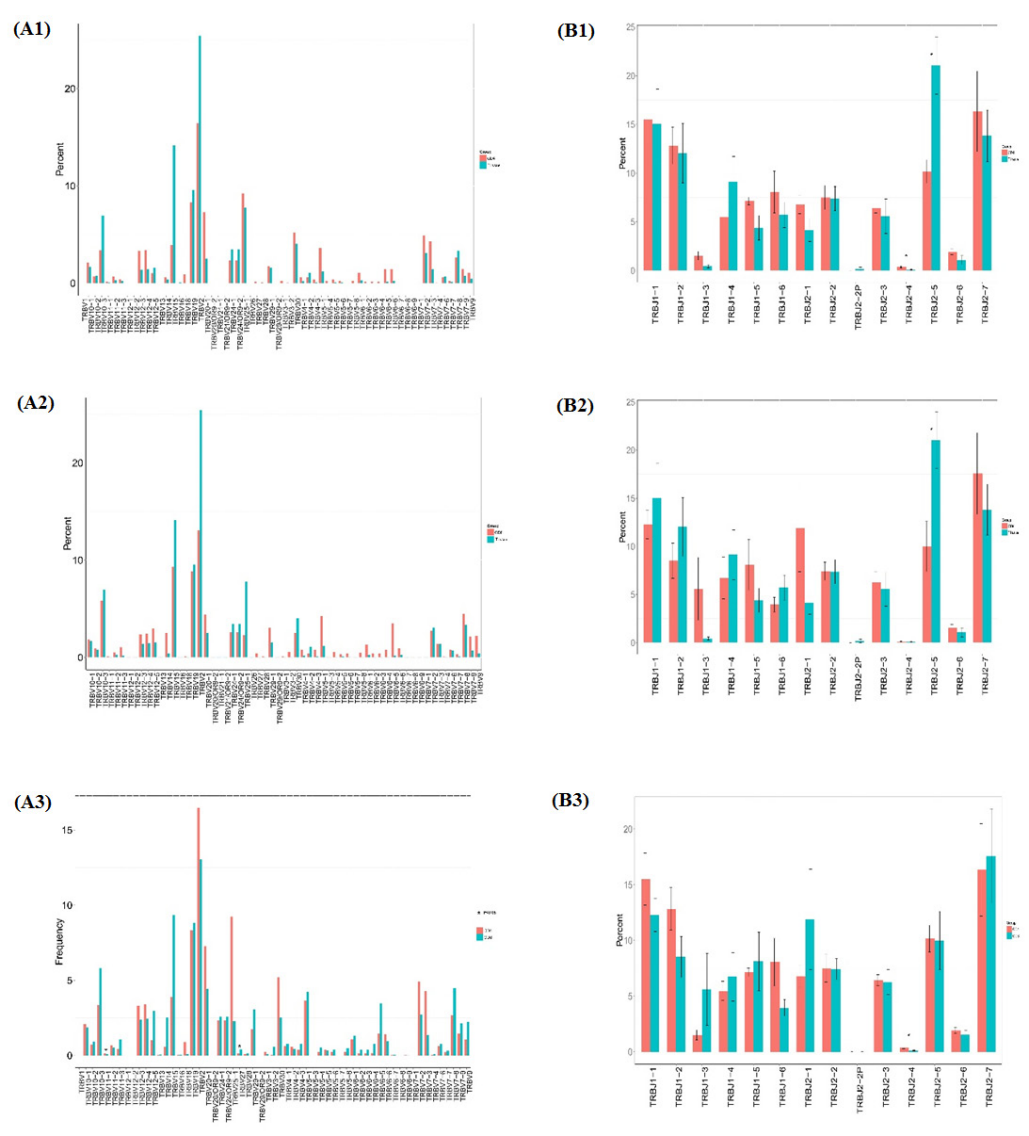
Comparison of TRBV gene and TRBJ gene usage in CD4+, CD8+, tissue groups (A1) Comparison of TRBV gene usage between CD4+ and tissue groups. (A2) Comparison of TRBV gene usage between CD8+ and tissue group. (A3) Comparison of TRBV gene usage in between CD4+ and CD8+ groups. (B1) Comparison of TRBJ gene usage between CD4+ and tissue groups. (B2) Comparison of CD8+ and tissue groups. (B3) Comparison of TRBJ gene usage between CD4+ and CD8+ groups.

We compared the TRBV and TRBJ repertoires of the 3 different sample type groups to analyze whether there is disease specific difference. In all the 62 TRBV genes, TRBV11-1 is significantly higher expressed in CD4+ group than in CD8+ group while TRBV27 is significantly poorly expressed in CD4+ group than in CD8+ group, but no significant difference was found when compare CD4+ group with tissue group or CD8+ group with tissue group. In all the 14 TRBJ genes, TRBJ2-4 is significant highly expressed in CD4+ group than in CD8+ group and highly expressed in CD4+ group than in sarcoidosis group, while TRBJ2-5 is significant poorly expressed in CD8+ group than in tissue group (Figure 3).

**Figure 4:**
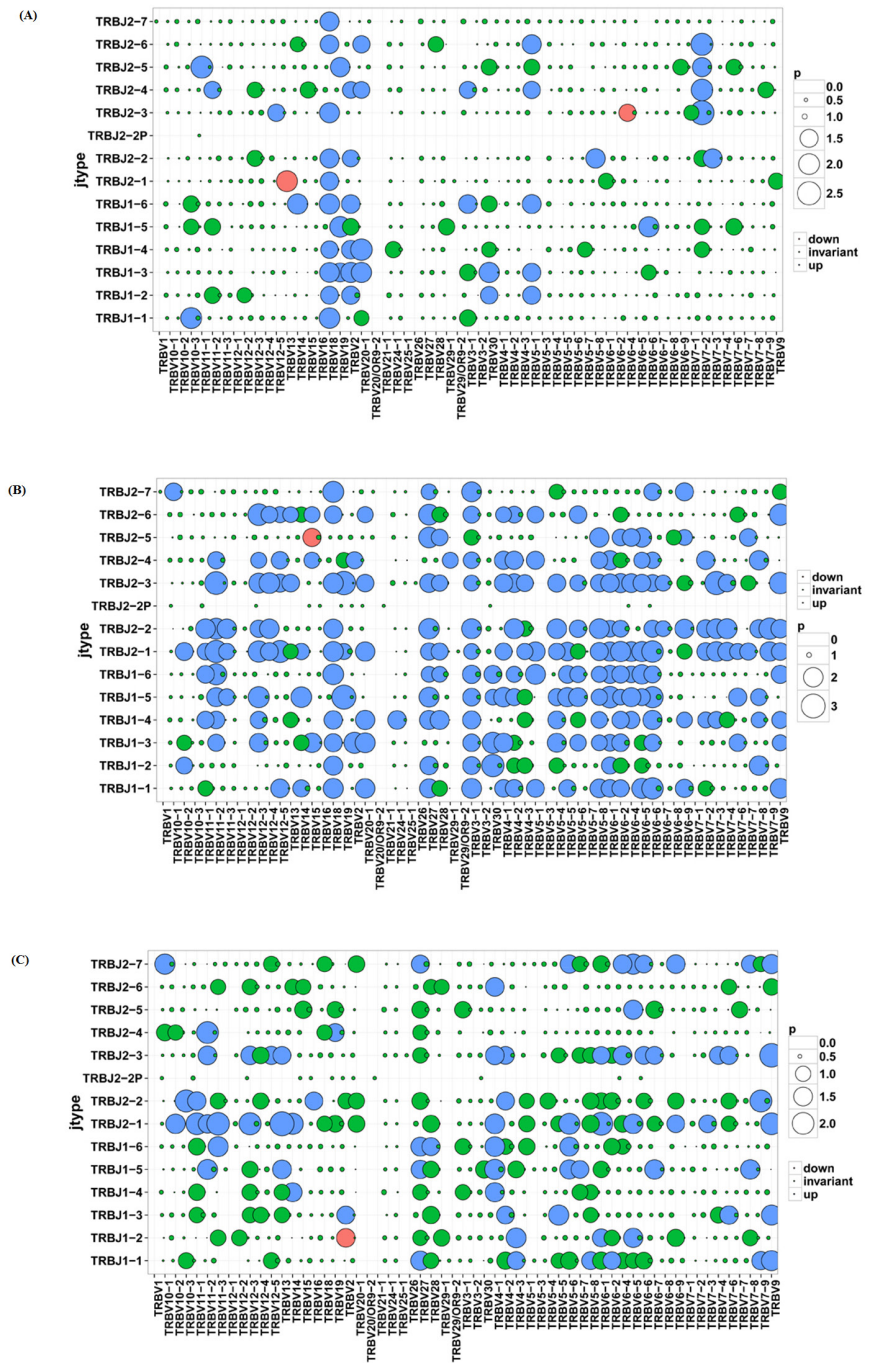
Comparison of V-J combinations among CD4+, CD8+ and tissue groups **A.** Comparison of V-J combinations between CD4+ and CD8+ group. **B.** Comparison of V-J combinations between CD4+ and tissue groups. **C.** Comparison of V-J combinations between CD8+ and tissue groups.

We also compared the relative frequencies of V-J combinations among three groups. In CD4+ group 44 VJ combinations pairs expressed higher usage and 2 VJ combination pairs showed lower usage than CD8+ group, while 101 VJ pairs showed higher usage and 1 VJ pair showed lower usage in CD4+ group than in sarcoidosis tissue group. In CD8+ group 74 VJ pairs showed higher usage and 1VJ pair showed lower usage than in tissue group (Figure 4). Significant differences were considered *p* < 0.05 by t-test.

### Diversity of TRBV and TRBJ repertories

To further understand the diversity of TCR CDR3 diversity, we calculated the length diversity Shannon index and sequence diversity Shannon index. In the situation of comparing TRBV length and sequence diversity Shannon index of CD4+, CD8+ and Tissue groups, we found the CD4+ group has the highest value in each comparison of TRBV length diversity, TRBV sequence diversity, TRBJ length diversity and TRBJ sequence diversity, although there is no statistics significance in each comparison (Supplementary Figure 2).

The CDR3 length distribution was analyzed by amino acids length and percentage in total sequences, the distribution was depicted and show in Supplementary Figure 3. The different amino acids length of TRBV gene CDR3 in CD4 and tissue groups ranged from 1-154, while it's 1-155 in CD8 group. The length distribution comparison among the 3 groups with Kruskal-Wallis testing showed significant difference (*p* < 0.05), and the AA length percentage distribution showed significant difference too(*p* < 0.05) Supplementary Figure 4.

The different amino acids length of TRBJ gene CDR3 in CD4 and CD8 groups ranged from 1-157, while the length in tissue group ranged from 1 to 146.The length distribution comparison among the 3 groups with Kruskal-Wallis testing showed no significant difference (p>0.05), and the AA length percentage distribution showed no significant difference too(p>0.05) (Supplementary Figure 4).

## DISCUSSION

The relationship between immune response and sarcoidosis has been studied for a long time. Part of the basic mechanism has been illuminated but the whole picture of immune repertoire influence was not clear. Since T cells play central roles in host protection against infectious pathogens and can contribute to the development of autoimmune and allergic diseases. There could be a potential relationship between sarcoidosis development and T cell immune response. Recently NGS is widely used in the study of immunology research and has made great progress. The usage of NGS, especially immune repertoire for the study of T cell immune response in sarcoidosis patients provided tremendous help. In this study, we compared different parameters of T cell repertoires in CD4+ lymphocytes samples (n = 7), CD8+ lymphocytes (n = 7) and tissues (n = 6) with a high through-put next generation sequencing and high quality control bio-informatics method. The CDR3 amino acid length is in Gaussian distribution in healthy situation, while skewed distribution would appear in disease situation when environmental or autoimmune pathogen attacks. We found the percentage of amino acid length 2, 16, 18 is extremely low in tissue groups with a total percent of 0, 1.78% and 0.1%, the percentage is significantly higher in CD4+ group (0.4%, 10.9%, 1.02%) and CD8+ group (0.07%, 8.9%, 1.67%). We believe this difference among 3 groups may highly relate to the different cellular components, especially in sarcoidosis tissues which are composed of lung cells, T-lymphocytes and macrophages.

Gini coefficient for inequality was a measurement for evenness of wealth distribution in economics, now introduced to describe the repertoire skewness. The Gini coefficient ranged from 0 to 1, the samples with better sequences distribution evenness would be calculated with smaller Gini coefficient [11]. We found the tissue group with the highest Gini coefficient, CD4+ group the lowest and medium CD8+ group. This suggests the tissue group has the most skewed sequences distribution, the possible explanation is the potential influence of sarcoidosis pathogen. In this study, we define the TCR clones with frequency above 0.5% of total reads in a sample were HECs. The comparison within the 3 groups showed the sarcoidosis group has the highest average HEC number (41.33) and average HEC ratio (66.18%), while the CD4+ group has the lowest average HEC number (7.85) and average HEC ratio (15.17%). The significant highly expanded HECs in tissue group are related to the high skewed distribution of TCRs in sarcoidosis tissues, and this is probably the result of direct impact of lung sarcoidosis pathogen.

In most T-cell immune response, the responding TCR repertoire consists of some private specific TCRs, which are observed in only one individual. On the contrary, public TCRs which are shared in different individuals are always considered as rare and unusual due to the low possibility of same antigen in different individuals [12]. In this study, we found a small portion of TCRs are shared by all samples in each group, with 2 nucleotide sequences (GTGCCTGAGTATGGTACAGCTAATGG CCGTCTTCATTTCCATGCGGTGCACTTTATG CGGACACTTCCTACAGGTAGCGTTGACCCTAAT, AGCCGACGT), 2 amino acid sequences (VPEYGTANGRLHFHAVHFMRTLPTGSVDPN, SSR) were public in all 20 samples. Besides, we also found an evident higher proportion of public TCRs in sarcoidosis tissue group (62 compare with 4 or 5). Despite the limited samples amount, the difference between tissue group and PMBC groups is potential targets for further study.

To further study the characteristics of the repertoire, we analyzed the usage of TRBV and TRBJ genes in different groups. In the study of 62 TRBV genes, TRBV11-1 and TRBV27 showed significant different in the comparison of CD4+ and CD8+ group. TRBJ2-4 and TRBJ2-5 gene were found differently express in the study of 14 TRBJ genes. The different usage of TRBV and TRBJ genes shows the comparable rearrangement variation in different tissue and blood groups. This also suggest the lack of randomly rearrangement TCR BV region in certain disease situation. In a study of T-cell reaction in Parvovirus B19 associated inflammatory cardiomyopathy, the dominance function of TRBV11 has been reported [13].

In conclusion, with the usage of HTS we succeeded in studying the hallmarks of sarcoidosis immune repertoire. Regarding the small sample amount, further study should concentrate on the study of basis mechanism of sarcoidosis, disease biomarker, potential therapeutic targets.

## MATERIALS AND METHODS

### Patients and controls

Whole blood samples from 7 sarcoidosis patients and lung tissue pathology samples from 6 of the 7 sarcoidosis patients were collected at The Second Medical College of Jinan University (Shenzhen People's Hospital), Shenzhen, China, and PBMCs were separated. Sarcoidosis was confirmed by pathologic diagnosis and in consistent with clinical evidence. 6 patients are female and 1 patient is male, the mean age is 42.1±8.91 years, ranging from 29 to 53 years old. Clinical symptoms include cough, chest tightness, palpitation, chest pain, and CT scan shows bilateral hilar and mediastinum multiple lymphadenopathy. 7 patients tested ACE level with a mean level of 60.77±18.06 U/L, ranging from 34 to 89 U/L. And the results of PPD test of these 7 patients are all negative. Written informed consent for study participation was obtained from all subjects. The use of PBMCs for further studies beyond routine diagnosis was approved by the local ethics committee. This study abides by the Helsinki Declaration on ethical principles for medical research involving human subjects.

### T cell isolation and DNA extraction

Informed consent was obtained from the blood donors. T cell isolations were performed using superparamagnetic polystyrene beads (Miltenyi) coated with monoclonal antibodies specific for T cells. DNA was prepared from 0.5 to 2 × 106 T cells from each sample, which was sufficient for analyzing the diversity of TCRV in the T cell subsets. DNA was extracted from PBMCs using GenFIND DNA (Agencourt, Beckman Coulter, Brea, CA, USA) extraction kits following the manufacturer's instructions.

### Multiplex-PCR amplification of the TCR CDR3 region

The TCR CDR3 region was defined according to International Immunogenetics collaboration, starting with the second conserved cysteine encoded by the 39 portion of the V gene segment and ending with the conserved phenylalanine encoded by the 59 portion of the J gene segment. To generate the template library for Genome Analyzer, a multiplex-PCR system was designed to amplify rearranged TCR CDR3 regions from genomic DNA using 45 forward primers, each specific to a functional TCR V segment, and 13 reverse primers, each specific to a TCR J segment. The forward and reverse primers contain, at their 5 ends, the universal forward and reverse primer sequences, respectively, which are compatible with GA2 cluster station solid-phase PCR. After amplification and agarose gel electrophoresis selection, the products were purified using QIAquick PCR Purification Kit. The final library was quantitated in two ways: by determining the average molecule length using the Agilent 2100 bioanalyzer instrument (Agilent DNA 1000 Reagents) and by real-time quantitative PCR (QPCR) (TaqMan Probe). The libraries were amplified with cBot to generate the cluster on the flow cell, and the amplified flow cell was pair-end sequenced using a Hiseq2000 instrument, with a read length of 100 as the most frequently used sequencing strategy.

### High-throughput sequencing and data analysis

The PCR products were sequenced using an Illumina Genome Analyzer, and the sequencing quality of these read were evaluated by the formula shows below. The quality of the HiSeq sequencing ranged from 0 to 40 and was used for filtering out low-quality reads. First, we filtered the raw data, including adapter contamination. Reads with an average quality score lower than 15 (Illumina 0-41 quality system) were removed, and the proportion of N bases was not more than 5% (sequences with higher values were also removed). Next, a few bases with low quality (lower than 10) were trimmed; the quality score was expected to be over 15 after trimming, and the remaining sequence length was expected to be more than 60 nt. After filtering, pair-end (PE) read pairs were merged into one contig sequence in two steps: 1, by aligning the tail parts of two sequences and assessing the identity (BGI developed software COPE v1.1.3), with at least 10 bases of overlap required and the overlapping section having 90% base match; 2, as different primers might result in sequences of different lengths, some might be very short (less than 100 bp) and will go thought all bases on the sequence, and such reads were merged by aligning the head part of the sequence (BGI developed software FqMerger). In this way, we obtained the merged contig sequences and the length distribution plot. Subsequently, we used miTCR, developed by MiLaboratory (http://mitcr.milaboratory.com/downloads/) for the alignment. This program has an automated adjustment mechanism for errors introduced by sequencing and PCR and will provide alignment statistical information, such as the CDR3 expression and INDEL. After alignment, we utilized the following method for the sequence structural analysis: (1) we calculated the number of each nucleotide and analyzed the proportion at each position; (2) according to the last position of the V gene, start site of the D gene, end site of the D gene, and start site of the J gene after alignment, we retrieve the INDEL (insertion and deletion) introduced during V-D-J recombination; (3) nucleotides were translated into amino acids. According to the identity of each sequence after alignment, the expression level of each clone was clear and calculated. The expression of each distinct DNA sequence, amino acid sequence and V-J combination was also identified. In addition, to measure the diversity of each sample, we calculated the distinct clone number, Simpson coefficient and Shannon-Waver coefficient based on different resolutions of distinct DNA sequences, amino acid sequences, and V-J combinations. The expression level of each sample was also calculated at different resolutions of distinct DNA sequence, amino acid sequence, and V-J combination. Moreover, we constructed the specific expression draft and plotted a heat-map according to the V-J combination profile. The diversity of the TCR repertoire was calculated based on the Simpson index of diversity (Ds) and the Shannon-Wiener index (H).

### Statistical analysis

Because of the small sample size in this study, the analysis of differences among the data groups was performed with the Mann-Whitney test. p Values less than 0.05 were considered significant. The statistical analyses were conducted with GraphPad Prism software (GraphPad Software, San Diego, CA, USA).

This work was supported by funds received from Shenzhen Science and Technology Foundation (Lung Sarcoidosis TCRß chain CDR3 immune repertoire high throughput sequencing study, project No. JCYJ20160422142121988).

## SUPPLEMENTARY MATERIALS FIGURES


