# Supplementary material for: Immunology repertoire study of pulmonary sarcoidosis T cells in CD4+, CD8+ PBMC and tissue
Source: Oncotarget. 2017 Aug 9;8(52):89515–26. doi: 10.18632/oncotarget.20085 (PMC5685688; doi:10.18632/oncotarget.20085)
Supplement: Supplementary file 1 [file oncotarget-08-89515-s001.pdf]

Immunology repertoire study of pulmonary sarcoidosis T cells in CD4+, CD8+ PBMC and tissue

SUPPLEMENTARY MATERIALS

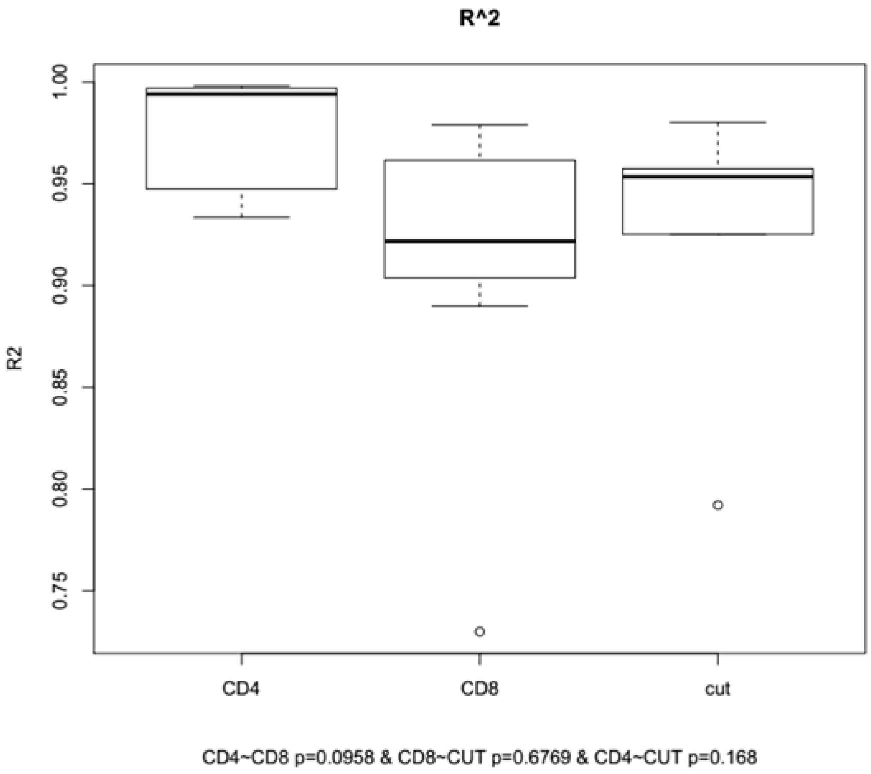

Supplimentary Figure 1: Gaussian distribution and the tissue group is worse fitted (no Sig.)

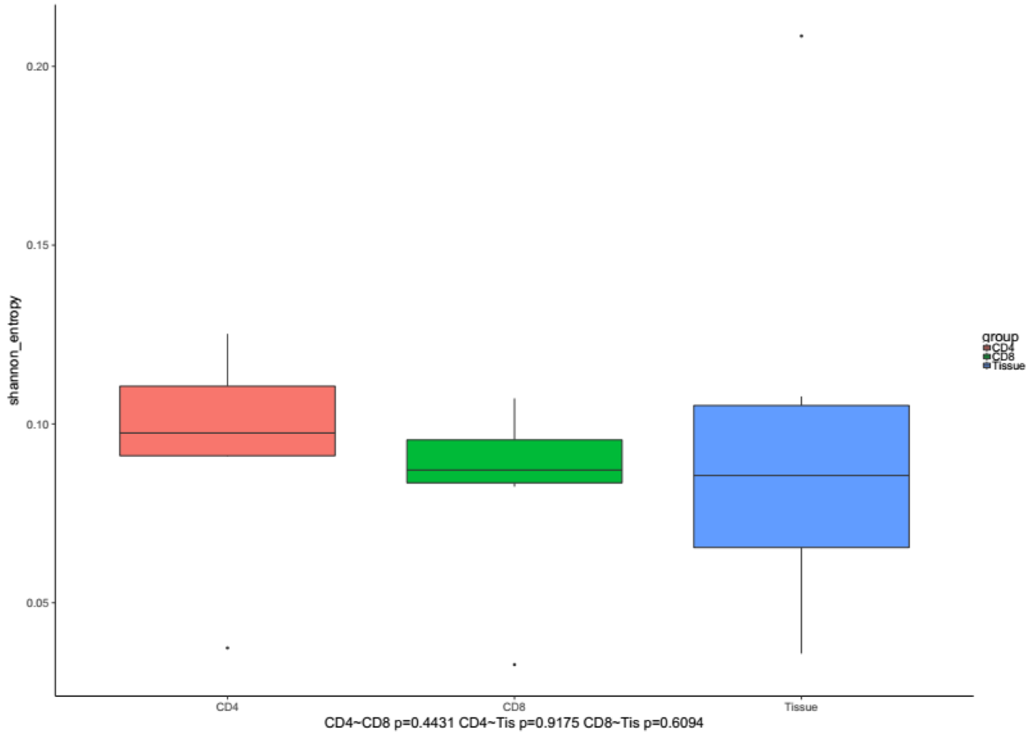

Supplimentary Figure 2A: Shannon entropy of TRBV gene CDR3 length diversity

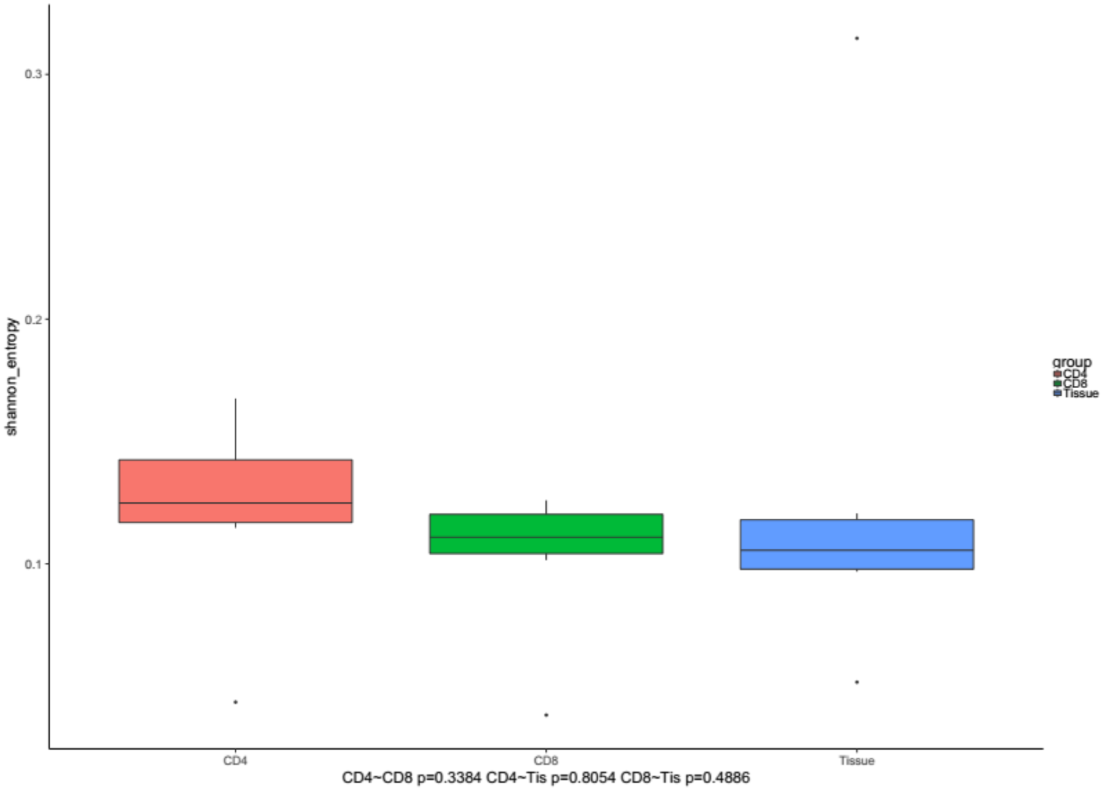

Supplementary Figure 2B: Shannon entropy of TRBV gene CDR3 sequence diversity

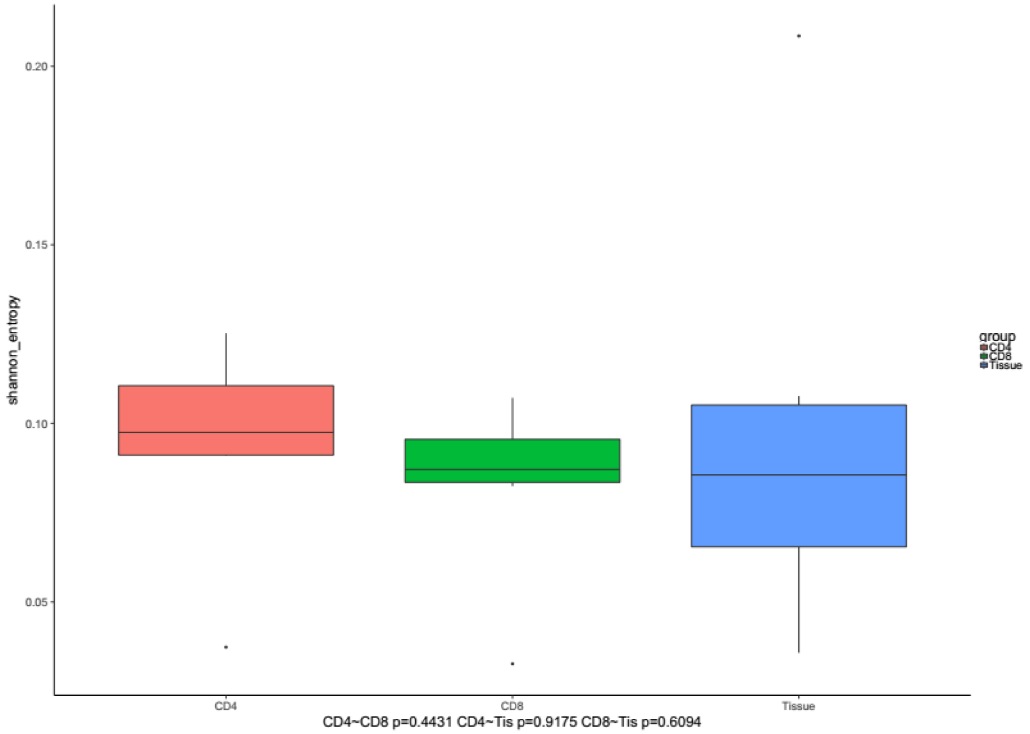

Supplementary Figure 2C: Shannon entropy of TRBV gene CDR3 length diversity

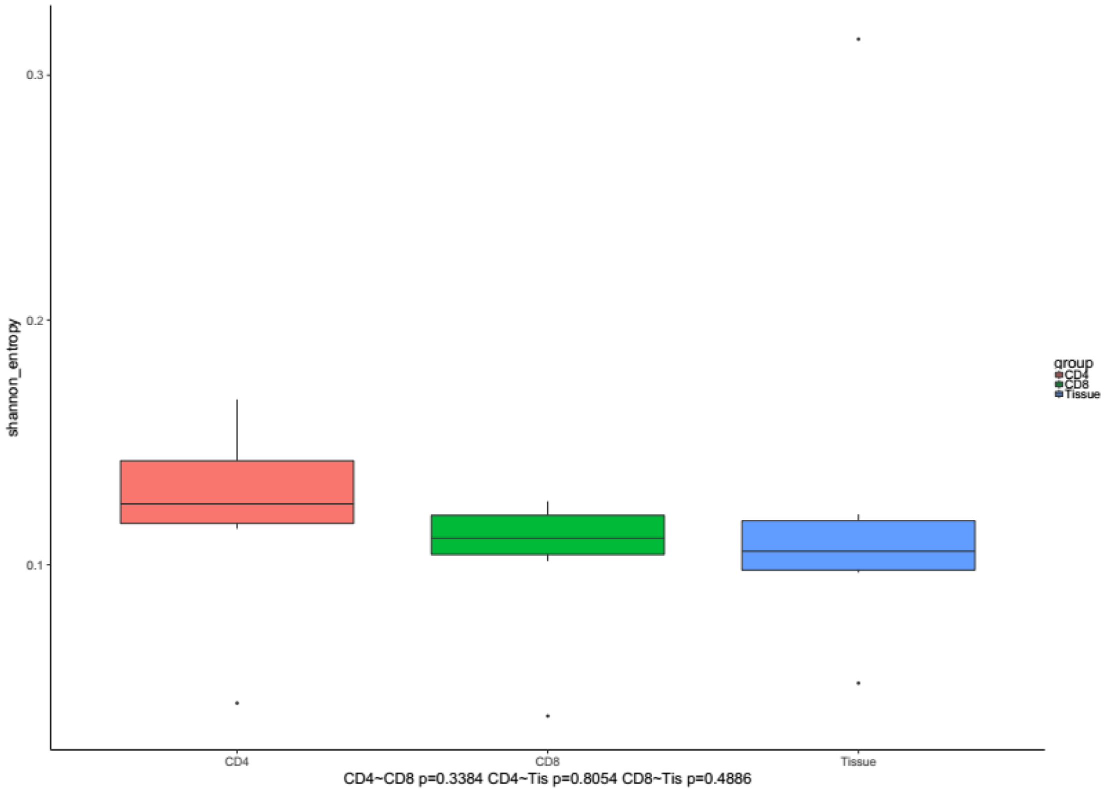

Supplementary Figure 2D: Shannon entropy of TRBV gene CDR3 sequence diversity

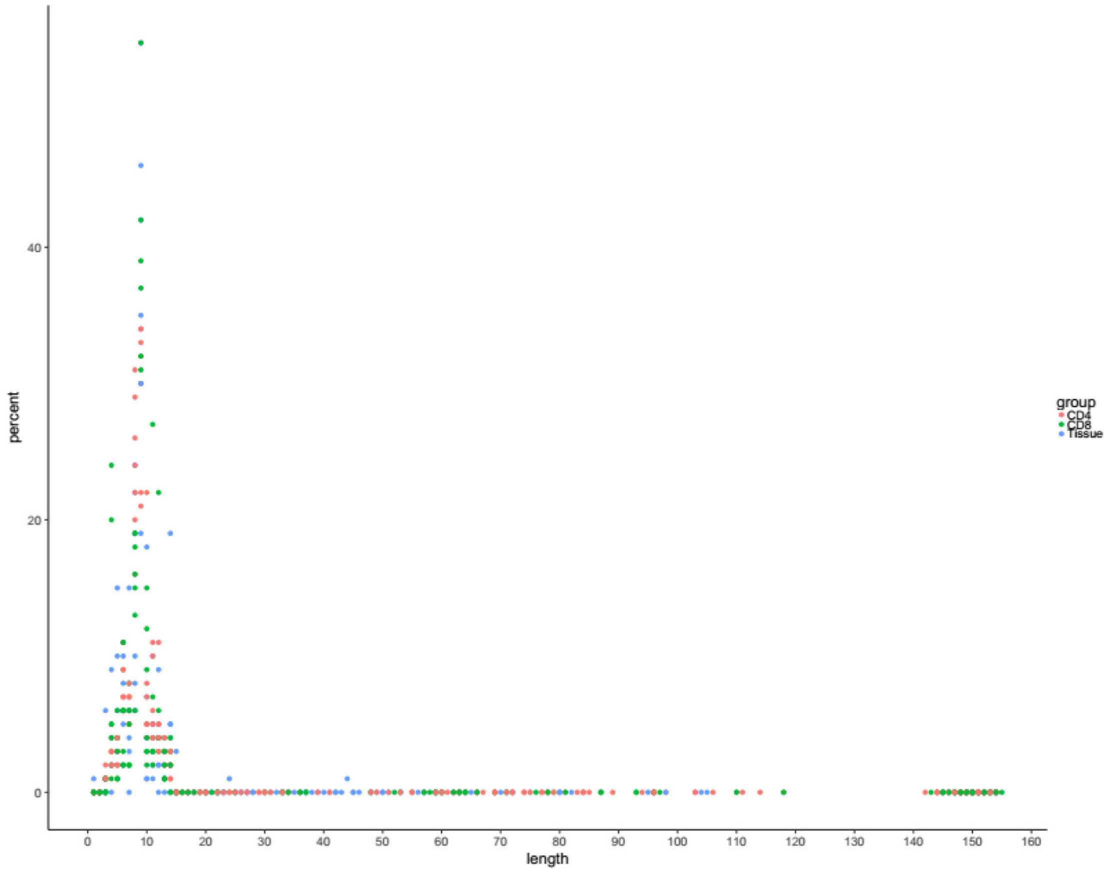

Supplementary Figure 3A: TRBV CDR3 length distribution of CD4+ group, CD8 group and tissue group.

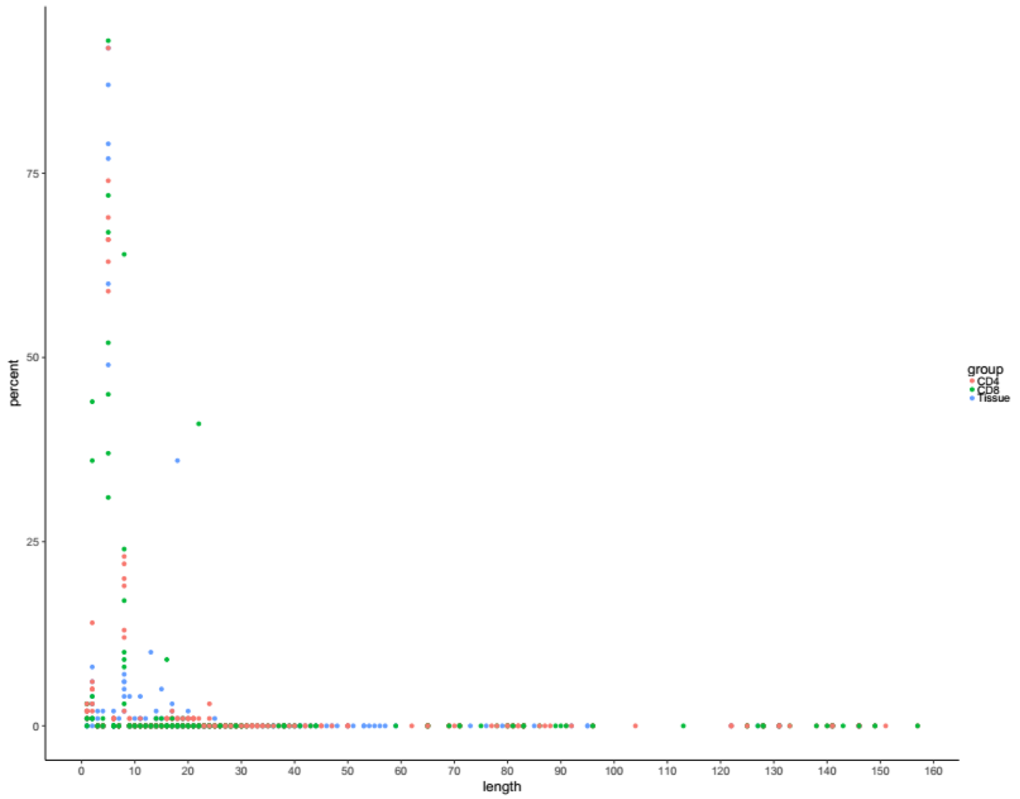

Supplementary Figure 3B: TRBJ CDR3 length distribution of CD4+ group, CD8 group and tissue group.

Independent-Samples Kruskal-Wallis Test

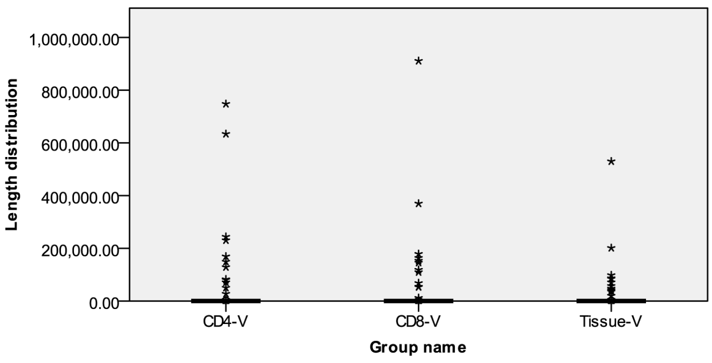

**Total N**	463
**Test Statistic**	7.416
**Degrees of Freedom**	2
**Asymptotic Sig. (2-sided test)**	.025

1. The test statistic is adjusted for ties.

Supplementary Figure 4A: TRBV CDR3 length distribution analysis;

Independent-Samples Kruskal-Wallis Test

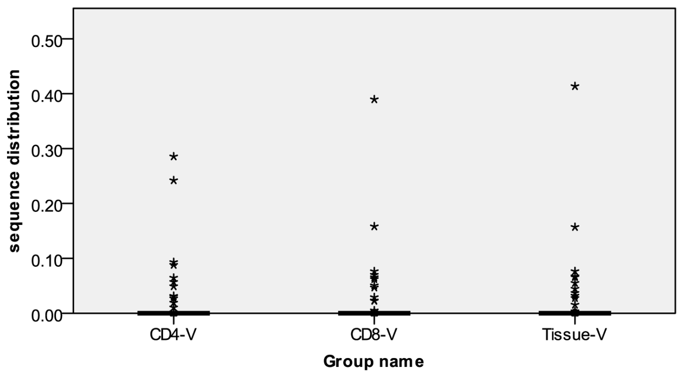

**Total N**	463
**Test Statistic**	8.505
**Degrees of Freedom**	2
**Asymptotic Sig. (2-sided test)**	.014

1. The test statistic is adjusted for ties.

Supplementary Figure 4B: TRBV CDR3 sequence distribution analysis.

Independent-Samples Kruskal-Wallis Test

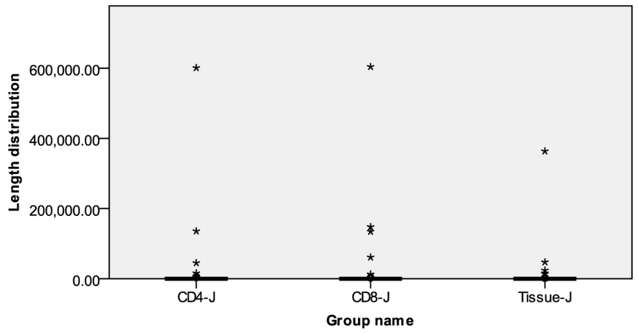

**Total N**	215
**Test Statistic**	.398
**Degrees of Freedom**	2
**Asymptotic Sig. (2-sided test)**	.820

1. The test statistic is adjusted for ties.
2. Multiple comparisons are not performed because the overall test does not show significant differences across samples.

Supplementary Figure 4C: TRBJ CDR3 Length distribution analysis.

Independent-Samples Kruskal-Wallis Test

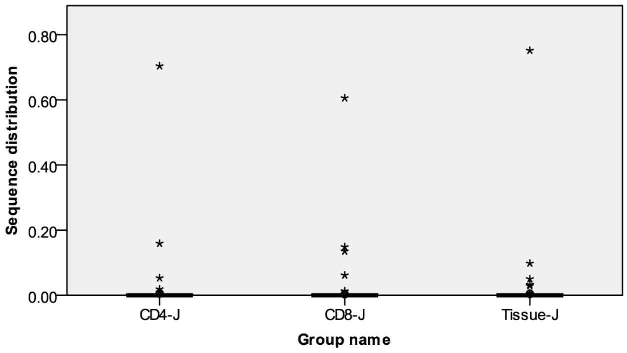

**Total N**	215
**Test Statistic**	4.844
**Degrees of Freedom**	2
**Asymptotic Sig. (2-sided test)**	.089

1. The test statistic is adjusted for ties.
2. Multiple comparisons are not performed because the overall test does not show significant differences across samples.

Supplementary Figure 4D. TRBJ CDR3 sequence distribution analysis;
